# Coming Together of Bayesian Inference and Skew Spherical Data

**DOI:** 10.3389/fdata.2021.769726

**Published:** 2022-02-08

**Authors:** Najmeh Nakhaei Rad, Andriette Bekker, Mohammad Arashi, Christophe Ley

**Affiliations:** ^1^Department of Mathematics and Statistics, Mashhad Branch, Islamic Azad University, Mashhad, Iran; ^2^DSI-NRF Centre of Excellence in Mathematical and Statistical Sciences (CoE-MaSS), Johannesburg, South Africa; ^3^Department of Statistics, University of Pretoria, Pretoria, South Africa; ^4^Department of Statistics, Faculty of Mathematical Sciences, Ferdowsi University of Mashhad, Mashhad, Iran; ^5^Department of Applied Mathematics, Computer Science and Statistics, Ghent University, Ghent, Belgium

**Keywords:** Fisher-von Mises-Langevin distribution, Gibbs sampling, MCMC method, skew-rotationally-symmetric distributions, slice sampler, spherical data, Wasserstein Impact Measure

## Abstract

This paper presents Bayesian directional data modeling via the skew-rotationally-symmetric Fisher-von Mises-Langevin (FvML) distribution. The prior distributions for the parameters are a pivotal building block in Bayesian analysis, therefore, the impact of the proposed priors will be quantified using the Wasserstein Impact Measure (WIM) to guide the practitioner in the implementation process. For the computation of the posterior, modifications of Gibbs and slice samplings are applied for generating samples. We demonstrate the applicability of our contribution via synthetic and real data analyses. Our investigation paves the way for Bayesian analysis of skew circular and spherical data.

## 1. Introduction

Big and complex data sets are collected from various scientific fields such as atmospheric environment, social science, psychological and biomedical studies, bioinformatics, epidemiology, digital imaging information, and machine learning, to name just a few. Big Data can refer to data with big volume or velocity, high-dimensional data (Ahmed, 2017), unstructured or unusual data (Härdle et al., 2018), complex data, etc. Therefore, there is a need for developing statistical techniques other than the traditional analytical frameworks to model, interpret, and use such data in different fields of science. Data with directions are categorized as unusual data that cannot be analyzed and modeled under the Cartesian coordinate system. With the aid of directional statistics, data science meets another level of analytical methods. In this research work, we focus on the analysis of complex directional data. Bayesian methods have received extensive attention in data science because prior information can be added to enhance modeling. Therefore, here, we consider Bayesian analysis of complex directional data.

A robust roadmap with the symmetric Fisher-von Mises-Langevin (FvML) distribution as the key element from a Bayesian perspective is briefly reviewed. According to Kikuchi's collection of directional data (Kikuchi, 1982), the first attention paid to Bayesian methods for directional data was in a paper by Mardia and El-Atoum (1976). They used the Bayesian approach to estimate the location parameter of the FvML distribution when the concentration parameter was known. The author Bagchi made several contributions in this area: (i) Bagchi (1988) formulated a conjugate prior for the mean direction and a non-informative prior for the concentration parameter of the von Mises distribution; (ii) Bagchi and Guttman (1988) focused on Bayesian inference for the multi-variate FvML distribution; (iii) Bagchi and Kadane (1991) derived the Bayes estimate for the cosine of the direction parameter of the von Mises distribution when the concentration parameter is known; and (iv) Bagchi (1994) developed empirical Bayesian techniques to estimate the mean direction of the FvML distribution (see also Guttorp and Lockhart, 1988; Dowe et al., 1996). Damien and Walker (1999) presented a full Bayesian inference for the von Mises distribution implementing a Gibbs sampler while (Rodrigues et al., 2000) provided an empirical or approximate Bayesian inference for the von Mises distribution. Nuñez-Antonio and Gutiérrez-Peña (2005) presented Bayesian analysis of the FvML distribution when all of the parameters were unknown, as well as an algorithm to generate samples from the posterior distribution based on a sampling-importance-resampling method. Muralidharan and Parikh (2007) provided Bayes estimates for both location and concentration parameters of the von Mises distribution. Bhattacharya and SenGupta (2009) presented Bayesian analysis of a generalized von Mises distribution introducing a new algorithm based on importance sampling and Markov chain Monte Carlo (MCMC) to draw samples from the posterior distribution. Mardia (2010) moved the attention to the bivariate von Mises distribution (on the torus) from a Bayesian viewpoint. Infinite mixtures of FvML distributions using standard conjugate priors for the parameters and Dirichlet priors for the mixing probabilities received attention from Bangert et al. (2010). Hornik and Grün (2013) defined conjugate and Jeffreys priors for the FvML distribution while Taghia et al. (2014) worked on Bayesian inference for the FvML mixture model. From 2017 and onwards the following contributions can be highlighted: Straub (2017) presented Bayesian analysis for the FvML distribution in 3D; Røge et al. (2017) presented Bayesian inference in the case of infinite FvML mixture model assumption; Mulder et al. (2020) provided Bayesian inference for mixtures of von Mises distributions using a reversible jump MCMC sampler and focused on non-informative priors. Lastly, the interested reader is referred to Pewsey and García-Portugués (2021) for Bayesian inference of other directional distributions.

Numerous directional data sets tend to show non-trivial features such as skewness. Therefore, the underlying distribution is not always symmetric, which emphasizes the focus on skewed directional distributions. This inspired us to investigate Bayesian analysis for the general class of skew-rotationally-symmetric distributions (Ley and Verdebout, 2017b), an asymmetric extension of all rotationally symmetric distributions, when the location, concentration, and skewness parameters are unknown.

In section 2, the skew-rotationally-symmetric distribution and special cases are described. The novel contribution is given in section 3 where the posterior distributions are obtained for the skew-FvML as the likelihood model, for four different scenarios of the prior distributions for the parameters of the model. Moreover, an algorithm is provided for generating samples from these posterior distributions. The impact of the priors is explored in section 4, by implementing the Wasserstein Impact Measure (WIM). In section 5, a synthetic data analysis is conducted to show the accuracy of the Bayes estimates based on the assumptions of the skew-FvML model. We demonstrate the applicability of the Bayesian framework for well known real datasets in section 6 for dimensions *p* = 2 and 3.

## 2. Skew-Rotationally-Symmetric Distributions

Most of the distributions on the unit hypersphere 𝕊^*p*−1^ = {***v*** ∈ ℝ^*p*^ : ***v***^⊤^***v*** = 1}, *p* ⩾ 2, share the common feature of being rotationally-symmetric about their location **μ** ∈ 𝕊^*p*−1^. The distribution of a random variable ***X*** ∈ 𝕊^*p*−1^ is said to be rotationally-symmetric about **μ** if for any orthogonal matrix ***O***_*p*×*p*_ satisfying ***Oμ*** = **μ** it is concluded that ***OX*** is equal in distribution to ***X***. The FvML distribution, the most common distribution in spherical studies, is obtained by conditioning on the *p*-variate normal distribution (see Mardia and Jupp, 2000; Ley and Verdebout, 2017a). Suppose ***X*** takes values on the non-linear manifold 𝕊^*p*−1^ and has the FvML distribution, then its probability density function (pdf) is given by


(1) $$\begin{array}{l} f\left(x;\mu ,\tau \right)=C\left(\tau \right)\exp\left(\tau \mu^{⊤}x\right),\;x,\mu \in {𝕊}^{p-1}, \end{array}$$

where


(2) $$\begin{array}{l} C\left(\tau \right)={\left(2\pi \right)}^{-p/2}\frac{\tau^{p/2-1}}{I_{p/2-1}\left(\tau \right)}, \end{array}$$

τ ⩾ 0 is the concentration parameter, and *I*_α_ is the modified Bessel function of order α. For τ = 0, (1) simplifies to the uniform distribution. If *p* = 2 in (1), it results in the von Mises distribution and when *p* = 3, the Fisher distribution (Fisher, 1953) is obtained.

However, in practice, all real-life phenomena cannot be represented by symmetric models. The interested reader is referred to Downs (2003) for medical research of heart disease diagnosis, Leong and Carlile (1998) for application in neurosciences, Shearman et al. (2000) for biological research on mammalian circadian rhythms, Mardia (2013) and Ameijeiras-Alonso and Ley (2020) for application in bioinformatics especially protein structure prediction, Fisher and Lee (1994) for some studies on wind direction, Ameijeiras-Alonso et al. (2021) for biomechanics studies, and Pewsey (2002) and Ley and Verdebout (2014) for animal movement studies.

Therefore, in this paper, the focus will be on the skew-rotationally-symmetric (SRS) distributions, introduced by Ley and Verdebout (2017b) as


(3) $$\begin{array}{l} f_{SRS}\left(x\right)=2f\left(x^{⊤}\mu \right)\Pi \left(\gamma^{⊤}{ϒ}_{\mu }^{⊤}x\right),\;x,\mu \in {𝕊}^{p-1}, \end{array}$$

where *f*(***x***^⊤^**μ**) is a rotationally-symmetric pdf about **μ** ∈ 𝕊^*p*−1^, Π : ℝ → [0, 1] is a monotone increasing function satisfying Π(−*t*) + Π(*t*) = 1 for all *t* ∈ ℝ, and ${ϒ}_{\mu }^{⊤}$ represents the semi-orthogonal matrix such that ${ϒ}_{\mu }{ϒ}_{\mu }^{⊤}=I_{p}-\mu \mu^{⊤}$ and ${ϒ}_{\mu }^{⊤}{ϒ}_{\mu }=I_{p-1}$, where ***I***_*p*_ is the *p* × *p* identity matrix. The parameter **γ** ∈ ℝ^*p*−1^ is a skewness parameter vector such that **γ** = **0** provides the symmetric pdf *f*(***x***^⊤^**μ**) and non-zero values of **γ** provide skewed pdfs. This construction allows using the full potential of existing rotationally-symmetric distributions by turning them into skewed versions.

Substituting (1) in (3) and letting ${ϒ}_{\mu }^{⊤}x=\left({\left(1-{\left(x^{⊤}\mu \right)}^{2}\right)}^{1/2}U_{\mu }\left(x\right)\right)$ with ***U***_**μ**_(***x***) the sign vector which is uniformly distributed on 𝕊^*p*−2^, the skew-FvML (*SFvML*) distribution is obtained as


(4) $$\begin{array}{l} f_{SFvML}\left(x;\mu ,\gamma ,\tau \right)\\ =2C\left(\tau \right)\exp\left(\tau x^{⊤}\mu \right)\Pi \left({\left(1-{\left(x^{⊤}\mu \right)}^{2}\right)}^{1/2}\gamma^{⊤}U_{\mu }\left(x\right)\right),\\ \;x,\mu \in {𝕊}^{p-1}, \end{array}$$

where *C*(τ) is defined in (2).

By using the standard cosine transformation


(5) $$\begin{array}{l} {\left(x_{1},x_{2},...,x_{p}\right)}^{⊤}\\ ={\left(\cos\theta_{1},\sin\theta_{1}\cos\theta_{2},...,\sin\theta_{1}...\sin\theta_{p-2}\sin\theta_{p-1}\right)}^{⊤}, \end{array}$$

and choosing ${ϒ}_{\mu }^{⊤}x=1\left(x⩾\mu \right)-1\left(x⩽\mu \right)$ in (4), for *p* = 2, the skew-von Mises (SvM) distribution follows as


(6) $$\begin{array}{l} f_{SvM}\left(\theta ;\mu ,\tau ,\gamma \right)=\frac{1}{\pi I_{0}\left(\tau \right)}\exp\left(\tau \cos\left(\theta -\mu \right)\right)\Pi \left(\gamma \sin\left(\theta -\mu \right)\right) \end{array}$$

where θ, μ ∈ (−π, π], τ > 0 and γ ∈ ℝ. Here, the scalar product ***x***^⊤^**μ** is cos(θ − μ). By choosing $\Pi \left(x\right)=\frac{1+x}{2}$, *x* ∈ [−1, 1], in (6), the sine-skewed von Mises distribution introduced by Abe and Pewsey (2011) is obtained where γ ∈ [−1, 1].

The following lemma can be used to generate a sample from the *SFvML* distribution.

Lemma 1. *(Ley and Verdebout, 2017b) Generate ***Y*** from the rotationally-symmetric FvML distribution in (1). Then for any uniformly distributed sign vector* ***U***_**μ**_(***Y***), ***U***_**μ**; Π_(***Y***) *is defined as*


$$\begin{array}{l} U_{\mu ;\Pi }\left(Y\right)=\{\begin{array}{cc} U_{\mu }\left(Y\right) & \;if\;U⩽\Pi \left({\left(1-{\left(Y^{⊤}\mu \right)}^{2}\right)}^{1/2}\gamma^{⊤}U_{\mu }\left(Y\right)\right),\\ -U_{\mu }\left(Y\right) & \;if\;U>\Pi \left({\left(1-{\left(Y^{⊤}\mu \right)}^{2}\right)}^{1/2}\gamma^{⊤}U_{\mu }\left(Y\right)\right), \end{array} \end{array}$$

*where U is uniformly distributed on* (0, 1) *and independent of ***Y***. Then the vector ***X*** with pdf (4) is obtained as*


$$\begin{array}{l} X=\left(Y^{⊤}\mu \right)\mu +{\left(1-{\left(Y^{⊤}\mu \right)}^{2}\right)}^{1/2}{ϒ}_{\mu }U_{\mu ;\Pi }\left(Y\right). \end{array}$$

In the next section, Bayesian inference with the *SFvML* distribution as the key element is presented with all location, concentration, and skewness parameters **μ**, τ, and **γ** unknown.

## 3. Methodology

Let ***X*** = (***X***_1_, ***X***_2_, ..., ***X***_*n*_) be a random sample of size *n* with pdf (4) where the standard normal cumulative density function (cdf) Φ replaces Π. The likelihood function is


(7) $$\begin{array}{l} L\left(\mu ,\gamma ,\tau |X\right)=2^{n}C^{n}\left(\tau \right)\exp\left(\tau \mu^{⊤}\overset{n}{\underset{i=1}{\sum }}x_{i}\right)\\ \;\overset{n}{\underset{i=1}{\prod }}\Phi \left({\left(1-{\left({x_{i}}^{⊤}\mu \right)}^{2}\right)}^{1/2}\gamma^{⊤}U_{\mu }\left(x_{i}\right)\right). \end{array}$$

Subsequently, four scenarios are presented to define the prior distributions for the location parameter **μ**, the concentration parameter τ, and the skewness parameter **γ**.

### 3.1. Prior Distributions

As above, let ***X*** denote a set of observations, and a generative model of the data be defined through a set of unknown parameters **Ω** = (**μ**, **γ**, τ) (see (7)). In this section the prior distributions for **Ω** = (**μ**, **γ**, τ) are outlined.

For the skewness vector **γ** the following prior distributions are proposed: (*i*) the multi-variate normal distribution with location parameter **ξ** and covariance matrix diag(**σ**), (*ii*) the multi-variate skew-normal distribution (Azzalini, 1985) with location parameter **ξ**, covariance matrix diag(**σ**) and skewness parameter **λ**, i.e.,


(8) $$\begin{array}{l} \pi_{1}\left(\gamma |\xi ,\text{diag}\left(\sigma \right)\right)\propto \overset{p-1}{\underset{i=1}{\prod }}\frac{1}{\sigma_{i}}\phi \left(\frac{\gamma_{i}-\xi_{i}}{\sigma_{i}}\right)\propto \phi_{p-1}\left(\gamma -\xi ;\text{diag}\left(\sigma \right)\right), \end{array}$$

and


(9) $$\begin{array}{l} \pi_{2}\left(\gamma |\xi ,\text{diag}\left(\sigma \right)\right),\lambda )\propto \overset{p-1}{\underset{i=1}{\prod }}\frac{1}{\sigma_{i}}\phi \left(\frac{\gamma_{i}-\xi_{i}}{\sigma_{i}}\right)\Phi \left(\lambda_{i}\frac{\gamma_{i}-\xi_{i}}{\sigma_{i}}\right)\\ \;\propto \phi_{p-1}\left(\gamma -\xi ;\text{diag}\left(\sigma \right)\right)\Phi_{p-1}\left(\gamma -\xi ;D\right), \end{array}$$

where $D=\text{diag}\left(\frac{\sigma_{1}}{\lambda_{1}},\cdot \cdot \cdot ,\frac{\sigma_{p-1}}{\lambda_{p-1}}\right)$, ξ_*i*_ ∈ ℝ, σ_*i*_ > 0, λ_*i*_ ∈ ℝ, ϕ is the standard normal pdf, ϕ_*n*_ and Φ_*n*_ are the pdf and cdf of the *n*-variate standard normal distribution, respectively. Next, the following priors for **μ** and τ are considered.

#### Case 1: Nuñśez-Antonio and Gutiérrez-Peñśa's Prior

In this case, we adopt the joint prior distribution of Nuñez-Antonio and Gutiérrez-Peña (2005) with direction parameter **μ**_0_, concentration parameters ζ and η for (**μ**, τ), i.e.,


(10) $$\begin{array}{l} \pi \left(\mu ,\tau |\mu_{0},\zeta ,\eta \right)\propto {\left(\frac{\tau^{p/2-1}}{I_{p/2-1}\left(\tau \right)}\right)}^{\zeta }\exp\left(\eta \tau \mu^{⊤}\mu_{0}\right), \end{array}$$

where $\mu_{0}\in {𝕊}^{p-1}$ and 0 < η < ζ. The normalization constant can only be obtained for some special cases. Straub (2017) computed the normalization constant of (10) for ζ = 1 and *p* = 3.

#### Case 2: FvML and Gamma Prior

In this case, the FvML and gamma distributions with parameters **μ**_0_, τ_0_, α, and β are proposed as priors for (**μ**, τ) (Muralidharan and Parikh, 2007), i.e.,


(11) $$\begin{array}{l} \pi \left(\mu ,\tau |\mu_{0},\tau_{0},\alpha ,\beta \right)\propto \exp\left(\tau_{0}\mu^{⊤}\mu_{0}\right)\tau^{\alpha -1}\exp\left(-\beta \tau \right), \end{array}$$

where τ_0_, α, β > 0 and **μ**_0_ ∈ [−π, π).

### 3.2. Posterior Distributions

Subsequently, the posterior distribution π(**Ω** ∣ ***X***) ∝ π(**Ω**)*L*(**Ω** ∣ ***X***) is determined for the different prior assumptions on **Ω** = (**μ**, **γ**, τ). Firstly, assume the prior distribution set up as described under case 1 and different prior distributions for the skewness parameter.

#### Scenario 1

Assume the prior distribution of the skewness parameter, **γ** is given by (8), then for given ***X*** the posterior distribution of (**μ**, **γ**, τ) can be obtained by using (7), (8), and (10) as


(12) $$\begin{array}{l} \pi \left(\mu ,\gamma ,\tau |X,\mu_{0},\zeta ,\eta ,\xi ,\sigma \right)\propto {\left(\frac{\tau^{p/2-1}}{I_{p/2-1}\left(\tau \right)}\right)}^{\zeta +n}\\ \exp\left(\tau \mu^{⊤}\left(\overset{n}{\underset{i=1}{\sum }}x_{i}+\eta \mu_{0}\right)\right)\phi_{p-1}\left(\gamma -\xi ;\text{diag}\left(\sigma \right)\right)\\ \;\times \overset{n}{\underset{i=1}{\prod }}\Phi \left({\left(1-{\left({x_{i}}^{⊤}\mu \right)}^{2}\right)}^{1/2}\gamma^{⊤}U_{\mu }\left(x_{i}\right)\right). \end{array}$$

The full conditionals for **μ**, **γ**, and τ are, respectively:


$$\begin{array}{l} \pi \left(\mu |\gamma ,\tau ,X\right)\propto \exp\left(\tau \mu^{⊤}\left(\overset{n}{\underset{i=1}{\sum }}x_{i}+\eta \mu_{0}\right)\right)\\ \;\overset{n}{\underset{i=1}{\prod }}\Phi \left({\left(1-{\left({x_{i}}^{⊤}\mu \right)}^{2}\right)}^{1/2}\gamma^{⊤}U_{\mu }\left(x_{i}\right)\right),\\ \pi \left(\gamma |\mu ,\tau ,X\right)\propto \phi_{p-1}\left(\gamma -\xi ;\text{diag}\left(\sigma \right)\right)\\ \;\overset{n}{\underset{i=1}{\prod }}\Phi \left({\left(1-{\left({x_{i}}^{⊤}\mu \right)}^{2}\right)}^{1/2}\gamma^{⊤}U_{\mu }\left(x_{i}\right)\right),\\ \pi \left(\tau |\mu ,\gamma ,X\right)\propto {\left(\frac{\tau^{p/2-1}}{I_{p/2-1}\left(\tau \right)}\right)}^{\zeta +n}\exp\left(\tau \mu^{⊤}\left(\overset{n}{\underset{i=1}{\sum }}x_{i}+\eta \mu_{0}\right)\right). \end{array}$$

#### Scenario 2

If we assume the prior distribution (9) for **γ**, the posterior distribution of (**μ**, **γ**, τ) can be obtained by using (7), (9), and (10) as follows


(13) $$\begin{array}{l} \pi \left(\mu ,\gamma ,\tau |X,\mu_{0},\zeta ,\eta ,\xi ,\sigma \right)\propto {\left(\frac{\tau^{p/2-1}}{I_{p/2-1}\left(\tau \right)}\right)}^{\zeta +n}\\ \exp\left(\tau \mu^{⊤}\left(\overset{n}{\underset{i=1}{\sum }}x_{i}+\eta \mu_{0}\right)\right)\phi_{p-1}\left(\gamma -\xi ;\text{diag}\left(\sigma \right)\right)\\ \;\times \overset{n}{\underset{i=1}{\prod }}\Phi \left({\left(1-{\left({x_{i}}^{⊤}\mu \right)}^{2}\right)}^{1/2}\gamma^{⊤}U_{\mu }\left(x_{i}\right)\right)\Phi_{p-1}\left(\gamma -\xi ;D\right). \end{array}$$

The full conditionals for **μ**, **γ**, and τ are, respectively:


$$\begin{array}{l} \pi \left(\mu |\gamma ,\tau ,X\right)\propto \exp\left(\tau \mu^{⊤}\left(\overset{n}{\underset{i=1}{\sum }}x_{i}+\eta \mu_{0}\right)\right)\\ \;\overset{n}{\underset{i=1}{\prod }}\Phi \left({\left(1-{\left({x_{i}}^{⊤}\mu \right)}^{2}\right)}^{1/2}\gamma^{⊤}U_{\mu }\left(x_{i}\right)\right),\\ \pi \left(\gamma |\mu ,\tau ,X\right)\propto \phi_{p-1}\left(\gamma -\xi ;\text{diag}\left(\sigma \right)\right)\Phi_{p-1}\left(\gamma -\xi ;D\right)\\ \;\times \overset{n}{\underset{i=1}{\prod }}\Phi \left({\left(1-{\left({x_{i}}^{⊤}\mu \right)}^{2}\right)}^{1/2}\gamma^{⊤}U_{\mu }\left(x_{i}\right)\right),\\ \pi \left(\tau |\mu ,\gamma ,X\right)\propto {\left(\frac{\tau^{p/2-1}}{I_{p/2-1}\left(\tau \right)}\right)}^{\zeta +n}\exp\left(\tau \mu^{⊤}\left(\overset{n}{\underset{i=1}{\sum }}x_{i}+\eta \mu_{0}\right)\right). \end{array}$$

#### Scenario 3

If the prior distribution of the skewness parameter **γ** is given by (8), for given ***X***, the posterior distribution of (**μ**, **γ**, τ), by using (7), (8), and (11), is


(14) $$\begin{array}{l} \pi \left(\mu ,\gamma ,\tau |X,\mu_{0},\tau_{0},\alpha ,\beta ,\xi ,\sigma \right)\\ \propto \frac{\tau^{np/2+\alpha -n-1}}{I_{p/2-1}^{n}\left(\tau \right)}\exp\left(\mu^{⊤}\left(\tau \overset{n}{\underset{i=1}{\sum }}x_{i}+\tau_{0}\mu_{0}\right)-\beta \tau \right)\\ \;\phi_{p-1}\left(\gamma -\xi ;\text{diag}\left(\sigma \right)\right)\\ \times \overset{n}{\underset{i=1}{\prod }}\Phi \left({\left(1-{\left({x_{i}}^{⊤}\mu \right)}^{2}\right)}^{1/2}\gamma^{⊤}U_{\mu }\left(x_{i}\right)\right). \end{array}$$

The full conditionals for **μ**, **γ**, and τ are, respectively:


$$\begin{array}{l} \pi \left(\mu |\gamma ,\tau ,X\right)\propto \exp\left(\mu^{⊤}\left(\tau \overset{n}{\underset{i=1}{\sum }}x_{i}+\tau_{0}\mu_{0}\right)\right)\\ \;\overset{n}{\underset{i=1}{\prod }}\Phi \left({\left(1-{\left({x_{i}}^{⊤}\mu \right)}^{2}\right)}^{1/2}\gamma^{⊤}U_{\mu }\left(x_{i}\right)\right),\\ \pi \left(\gamma |\mu ,\tau ,X\right)\propto \phi_{p-1}\left(\gamma -\xi ;\text{diag}\left(\sigma \right)\right)\\ \;\overset{n}{\underset{i=1}{\prod }}\Phi \left({\left(1-{\left({x_{i}}^{⊤}\mu \right)}^{2}\right)}^{1/2}\gamma^{⊤}U_{\mu }\left(x_{i}\right)\right),\\ \pi \left(\tau |\mu ,\gamma ,X\right)\propto \frac{\tau^{np/2+\alpha -n-1}}{I_{p/2-1}^{n}\left(\tau \right)}\exp\left(\tau \left(\mu^{⊤}\overset{n}{\underset{i=1}{\sum }}x_{i}-\beta \right)\right). \end{array}$$

#### Scenario 4

When the prior distribution of **γ** is the skew-normal distribution in (9), the posterior distribution of (**μ**, **γ**, τ) by using (7), (9), and (11) is


(15) $$\begin{array}{l} \pi \left(\mu ,\gamma ,\tau |X,\mu_{0},\tau_{0},\alpha ,\beta ,\xi ,\sigma \right)\\ \propto \frac{\tau^{np/2+\alpha -n-1}}{I_{p/2-1}^{n}\left(\tau \right)}\exp\left(\mu^{⊤}\left(\tau \overset{n}{\underset{i=1}{\sum }}x_{i}+\tau_{0}\mu_{0}\right)-\beta \tau \right)\\ \;\phi_{p-1}\left(\gamma -\xi ;\text{diag}\left(\sigma \right)\right)\\ \times \overset{n}{\underset{i=1}{\prod }}\Phi \left({\left(1-{\left({x_{i}}^{⊤}\mu \right)}^{2}\right)}^{1/2}\gamma^{⊤}U_{\mu }\left(x_{i}\right)\right)\Phi_{p-1}\left(\gamma -\xi ;D\right). \end{array}$$

The full conditionals for **μ**, **γ**, and τ are, respectively:


$$\begin{array}{l} \pi \left(\mu |\gamma ,\tau ,X\right)\propto \exp\left(\mu^{⊤}\left(\tau \overset{n}{\underset{i=1}{\sum }}x_{i}+\tau_{0}\mu_{0}\right)\right)\\ \;\overset{n}{\underset{i=1}{\prod }}\Phi \left({\left(1-{\left({x_{i}}^{⊤}\mu \right)}^{2}\right)}^{1/2}\gamma^{⊤}U_{\mu }\left(x_{i}\right)\right),\\ \pi \left(\gamma |\mu ,\tau ,X\right)\propto \phi_{p-1}\left(\gamma -\xi ;\text{diag}\left(\sigma \right)\right)\Phi_{p-1}\left(\gamma -\xi ;D\right)\\ \;\times \overset{n}{\underset{i=1}{\prod }}\Phi \left({\left(1-{\left({x_{i}}^{⊤}\mu \right)}^{2}\right)}^{1/2}\gamma^{⊤}U_{\mu }\left(x_{i}\right)\right),\\ \pi \left(\tau |\mu ,\gamma ,X\right)\propto \frac{\tau^{np/2+\alpha -n-1}}{I_{p/2-1}^{n}\left(\tau \right)}\exp\left(\tau \left(\mu^{⊤}\overset{n}{\underset{i=1}{\sum }}x_{i}-\beta \right)\right). \end{array}$$

### 3.3. Sampling From the Posterior Distributions

A general algorithm is presented to obtain the Bayes estimates of the parameters **Ω** = (**μ**, **γ**, τ) based on the modified sampling-resampling method (Smith and Gelfand, 1992) and modified Gibbs sampling.

After a sufficient burn-in period, the generated sample ((**μ**_1_, **γ**_1_, τ_1_), (**μ**_2_, **γ**_2_, τ_2_), ..., (**μ**_*N*_, **γ**_*N*_, τ_*N*_)) is approximately distributed according to the posterior distribution of **Ω** = (**μ**, **γ**, τ). As can be seen in Algorithm 1, it is sufficient to generate samples of size *k* from prior distributions of (**μ**, **γ**, τ) which is one of the advantages of this algorithm. By increasing *N* and *k* in Algorithm 1 the approximation increases. When the joint prior distributions are not independent, Algorithm 1 still has a good performance (Muralidharan and Parikh, 2007). For the joint prior of (**μ**, τ) in (10), the slice sampler can be used (see McElreath, 2020).

**Algorithm 1:** Steps to generate samples from the posteriors by using priors and full conditionals.

1:	Set the initial values **Ω**^(0)^ = (**μ**^(0)^, **γ**^(0)^, τ^(0)^) and fix the sample size as *B* + *N*, where the first *B* samples are burn-in.
2:	Simulate **μ**_1_, **μ**_2_, ..., **μ**_*k*_, a sample of size *k*, from π(**μ**), the prior distribution of **μ**.
3:	Let $\rho_{1i}=\frac{\pi \left(\mu_{i}|X,\gamma^{\left(0\right)},\tau^{\left(0\right)}\right)}{\pi \left(\mu_{i}\right)}$ for *i* = 1, 2, ..., *k*, where π(**μ** ∣ ***X***, **γ**, τ) is the full conditional of **μ**.
4:	Select **μ**^(1)^ corresponding to maxρ_1*i*_, *i* = 1, 2, ..., *k*.
5:	Simulate τ_1_, τ_2_, ..., τ_*k*_ from π(τ), the prior distribution of τ.
6:	Let $\rho_{2i}=\frac{\pi \left(\tau_{i}|X,\mu^{\left(1\right)},\gamma^{\left(0\right)}\right)}{\pi \left(\tau_{i}\right)}$ for *i* = 1, 2, ..., *k*, where π(τ ∣ ***X***, **μ**, **γ**) is the full conditional of τ.
7:	Select τ^(1)^ corresponding to maxρ_2*i*_, *i* = 1, 2, ..., *k*.
8:	Simulate **γ**_1_, **γ**_2_, ..., **γ**_*k*_ from π(**γ**), the prior distribution of **γ**.
9:	Let $\rho_{3i}=\frac{\pi \left(\gamma_{i}|X,\mu^{\left(1\right)},\tau^{\left(1\right)}\right)}{\pi \left(\gamma_{i}\right)}$ for *i* = 1, 2, ..., *k*, where π(**γ** ∣ ***X***, **μ**, τ) is the full conditional of **γ**.
10:	Select **γ**^(1)^ corresponding to maxρ_3*i*_, *i* = 1, 2, ..., *k*.
11:	Repeat Steps 1-10 with **Ω**^(1)^ = (**μ**^(1)^, **γ**^(1)^, τ^(1)^) to obtain **Ω**^(2)^ = (**μ**^(2)^, **γ**^(2)^, τ^(2)^) and continue until **Ω**^(*B* + *N*)^ is obtained.

## 4. The Wasserstein Impact Measure

The prior distributions are a crucial part in Bayesian analysis. If the sample size is small, or available data provide only indirect information about the parameters of interest, the prior distribution becomes more important (Carlin and Louis, 2008). Different criteria can be used for prior selection, we refer the reader to Vehtari et al. (2017). Ghaderinezhad et al. (2022) implemented the Wasserstein Impact Measure (WIM) as a measure of the impact of the choice of the prior in a Bayesian approach. In fact it is a convenient way for quantifying prior impact which will help us to choose between two or more priors in a given situation. Suppose **Ω** is the vector of parameters and *F*_1_(.) and *F*_2_(.) are two cumulative distribution functions (cdfs) of two posterior distributions π_1_(**Ω**|.) and π_2_(**Ω**|.). The Wasserstein distance between these two posteriors related to two different prior sets is obtained as follows:


(16) $$\begin{array}{l} d_{W}\left(\pi_{1},\pi_{2}\right)=\int_{D_{\Omega }}|F_{1}\left(\Omega ;X\right)-F_{2}\left(\Omega ;X\right)|d\Omega , \end{array}$$

with *D*_**Ω**_ the domain of all possible values of **Ω**. The Wasserstein distance between two posteriors indicates, at any finite sample size *n*, how close the posterior distributions are and how similar the related inference will be. This is particularly interesting when considering a simple vs. a complicated, computationally intense prior; if the WIM between them is small, then one can safely use the simpler version. When *n* → ∞ the distance tends to 0.

In this section, a simulation study is conducted to compare the different sets of proposed priors in section 3 for *p* = 2 using this measure. Since the cdfs of the posteriors in (12)–(15) are not computable, Algorithm 1 and Monte Carlo integration are used to obtain the Wasserstein distance. Also, the transport package (Schuhmacher et al., 2020) in the *R* software offers functions for computing the Wasserstein distance between two sets of samples from different distributions. Most of the functions in this package have been designed for data with two or higher dimensions. For various combinations of the parameters we draw 200 random observations from the *SvM* in (6).

To compare the impact of the normal distribution and skew-normal distribution in (8) and (9) (for *p* = 2) as priors for the skewness parameter γ, the following steps were performed:

1. μ and τ were considered as known parameters.
2. For the unknown skewness parameter γ, the *N*(0, 5) and *SN*(0, 5, λ) with λ = −3, −2, −1, 1, 2, 3 were considered as priors.
3. For a generated sample from (6) (the skewing function is the standard normal cdf), with μ = 3, τ = 1 and γ = 5, the posteriors π_1_(γ|.) and π_2_(γ|.) emanating from *N*(0, 5) and *SN*(0, 5, λ), respectively, were considered.
4. The posteriors π_1_(γ|.) and π_2_(γ|.) were sampled using Algorithm 1, for *n* = 10, 15, 20, 25, 30, 35, 40, 50, 100.
5. The Wasserstein distance between the posteriors π_1_(γ|.) and π_2_(γ|.) was estimated, using the transport package and Monte Carlo method with 1, 000 repetitions.

**Figure 1:**
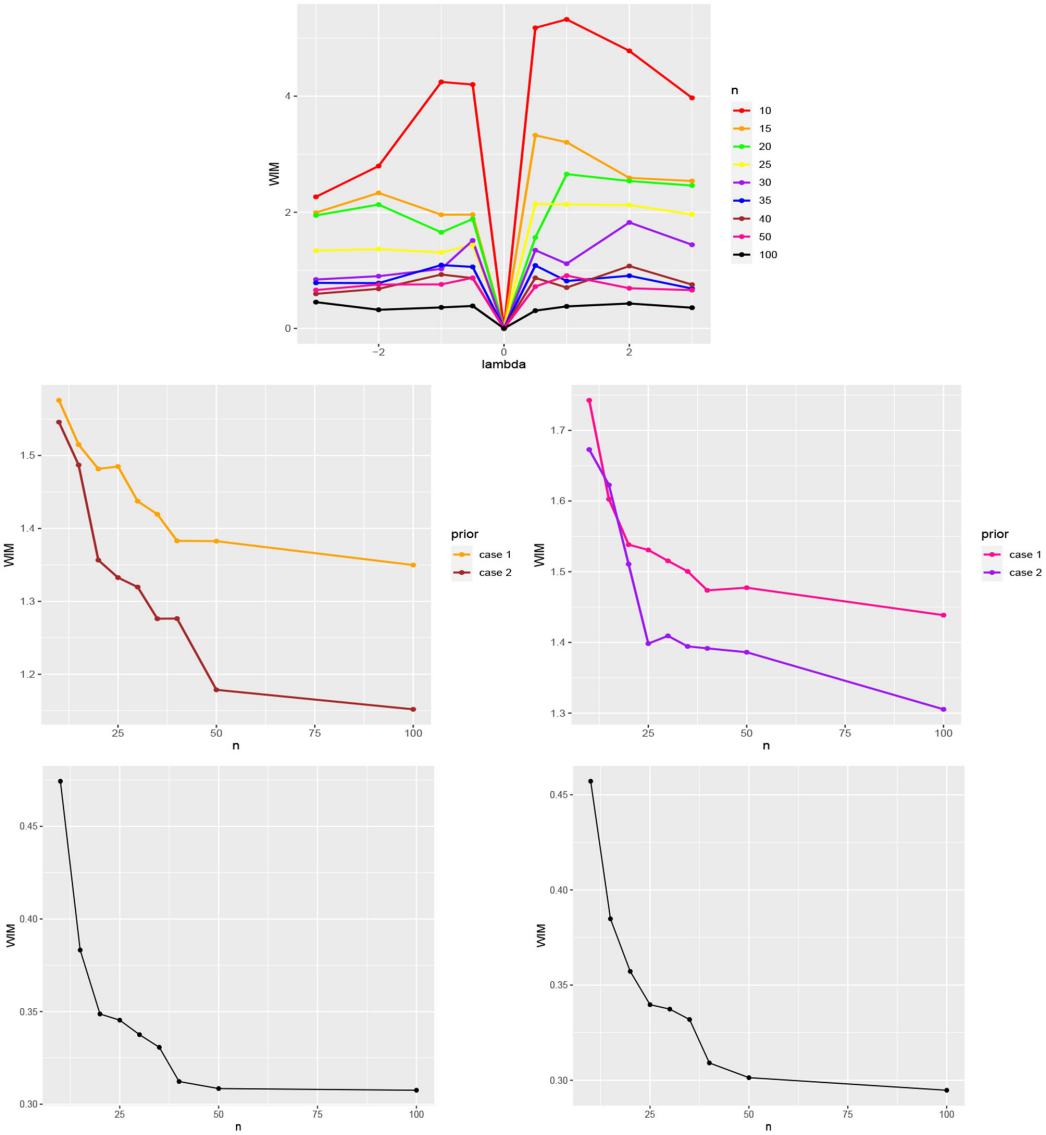
**(Top)** WIM values for comparing the normal and skew-normal distributions as priors for the skewness parameter γ for different values of λ and *n*. **(Middle)** the Wasserstein distance between the posteriors π_1_ and π_2_ (case 1) and also π_1_ and π_3_ (case 2) for μ = 2, τ = 1, γ = −1 (left) and μ = 3, τ = 0.6, γ = 1 (right) and different values of *n*. **(Bottom)** the Wasserstein distance between the posteriors π_2_ and π_3_ for μ = 2, τ = 1, γ = −1 (left) and μ = 3, τ = 0.6, γ = 1 (right) and different values of *n*.

Figure 1 (top) shows the calculated Wasserstein distance for different values of λ and *n*. As expected,

- when λ is close to 0, there are no nearly differences between the posteriors π_1_(**Ω**|.) and π_2_(**Ω**|.) for different values of *n*.
- by increasing *n*, the Wasserstein distance decreases. Hence, for large values of *n* the difference between the posteriors is minimal.

To illustrate the impact of the prior selection for μ and τ the following approach was followed. Assume the normal distribution as the prior for the skewness parameter γ. The posteriors π_2_(**Ω**|.) and π_3_(**Ω**|.), emanating from the informative priors (10) (case 1) and (11) (case 2) were compared with the posterior resulting from the non-informative prior μ ~ *Uniform*(0, 2π) and π(τ) ∝ 1, denoted by π_1_(**Ω**|.). The posteriors were sampled using Algorithm 1 for *n* = 10, 15, 20, 25, 30, 35, 40, 50, 100. The Wasserstein distances were calculated between them with the transport package and Monte Carlo method with 1, 000 repetitions.

Figure 1 (middle) illustrates the obtained Wasserstein distance between the posteriors π_1_(**Ω**|.) and π_2_(**Ω**|.) (case 1) and between π_1_(**Ω**|.) and π_3_(**Ω**|.) (case 2) for μ = 2, τ = 1, γ = −1 (left) and μ = 3, τ = 0.6, γ = 1 (right). Figure 1 (bottom) shows the Wasserstein distance between the posteriors π_2_(**Ω**|.) and π_3_(**Ω**|.) for μ = 2, τ = 1, γ = −1 (left) and μ = 3, τ = 0.6, γ = 1 (right), respectively. From Figure 1 (middle and bottom) the following observations can be obtained:

- The impact of the informative priors (10) (case 1) and (11) (case 2) for μ and τ is clearly visible in comparison with the assumed non-informative priors.
- Comparatively, the posterior resulting from prior (11) (case 2) is closer to the non-informative priors.
- By increasing *n*, the posteriors resulting from the informative priors (10) (case 1) and (11) (case 2) tend to the case of non-informative priors.
- There is less difference between the informative priors (10) (case 1) and (11) (case 2), than with respect to the non-informative priors.
- By increasing *n*, the posteriors resulting from the informative priors (10) (case 1) and (11) (case 2) approach each other.

We can thus conclude that from moderate sample sizes on, both priors for all three parameters are rather similar (hence one could use the less computationally intense of both priors), but differ clearly from a non-informative one. In order to judge how large the obtained WIM values actually are, bootstrap re-sampling could be done with the original data; we leave this for future research. Our analysis here is also limited to the chosen parameter values; more simulations need to be done to get a complete picture.

A similar analysis can be performed for *p* = 3.

## 5. Synthetic Data Analysis

In this section, to assess the performance of the Bayesian approach for obtaining the estimates of **Ω** = (μ, τ, γ), a synthetic data analysis was conducted to obtain the Bayes estimates of the parameters of the *SvM* distribution (6). We generated samples of size *N* = 20, 50, 100, 500 from the posterior distributions (12)-(15) (scenarios 1–4) with a burn-in period of 5,000 and *k* = 500, using Algorithm 1 (the values of these parameters are written down in the respective tables). It is noteworthy that steps 2–7 in Algorithm 1 are combined for scenarios 1 and 2. Bayes estimates of the parameters μ, τ and γ were obtained under the squared error, absolute error and zero-one loss functions by calculating mean, median, and mode of the generated samples, respectively.

The results for *p* = 2 and *p* = 3 including the sample mean, standard deviation, quartiles, and mode of the posterior distribution are summarized in Table 1 for each of the scenarios. As can be seen in Table 1 the obtained Bayes estimates are close to the actual values of the parameters. In addition, for small sample sizes our proposed Bayesian approach still provides accurate estimates.

**Table 1:** Bayes estimates of parameters for *p* = 2 based on scenario 1 with prior parameters μ_0_ = 1, τ_0_ = 9, α = 0.5, β = 5, ξ = −4, σ = 1, scenario 2 with prior parameters μ_0_ = 1, ζ = 10, η = 0.5, ξ = 0.5, σ = 0.5, λ = −1, scenario 3 with prior parameters μ_0_ = 1, τ_0_ = 9, α = 0.5, β = 5, ξ = −4, σ = 1, and scenario 4 with prior parameters μ_0_ = 0.5, τ_0_ = 0.01, α = 0.5, β = 9, ξ = 0.5, σ = 0.5, λ = −2 and for *p* = 3 based on scenario 3 with prior parameters μ_0_1__ = 1, τ_0_1__ = 5, μ_0_2__ = 2, τ_0_2__ = 9, α = 12, β = 2, ξ_1_ = 1, σ_1_ = 2, ξ_1_ = −2, and σ_1_ = 2.

	**Parameter**	**Actual**	**Mean**	**sd**	***Q*_1_**	**Median**	***Q*_**3**_**	**Mode**
Scenario 1 (*p* = 2)								
	μ	2.0	2.0931	0.1498	1.8640	2.0719	2.3978	1.9445
*n* = 500	τ	1.0	1.0089	0.2867	0.3646	1.0031	1.5859	1.0884
	γ	−1.0	−0.9848	0.0019	−0.9879	−0.9850	−0.9808	−0.9832
	μ	2.0	2.0005	0.2908	1.3715	1.9963	2.5974	1.9910
*n* = 100	τ	1.0	1.0247	0.2975	0.3902	1.0376	1.5037	1.1037
	γ	−1.0	−0.9749	0.0044	−0.9825	−0.9749	−0.9660	−0.9745
	μ	2.0	1.9953	0.3032	1.4256	1.9830	2.6032	1.9127
*n* = 50	τ	1.0	1.0617	0.3153	0.3991	1.1056	1.5552	0.9032
	γ	−1.0	−0.9755	0.0041	−0.9820	−0.9760	−0.9655	−0.9787
	μ	2.0	1.9386	0.2645	1.5802	1.8836	2.4149	2.1606
*n* = 20	τ	1.0	1.0367	0.2317	0.5845	1.0376	1.4549	1.1174
	γ	−1.0	−0.9753	0.0036	−0.9806	−0.9753	−0.9694	−0.9734
Scenario 2 (*p* = 2)								
	μ	3.0	2.9830	0.0598	2.8158	2.9821	3.0978	2.9747
*n* = 500	τ	0.6	0.5715	0.1532	0.2072	0.5661	0.8942	0.5404
	γ	1.0	1.0269	0.0969	0.8801	1.0058	1.2686	0.9833
	μ	3.0	3.1035	0.1040	2.9108	3.0964	3.3410	3.1665
*n* = 100	τ	0.6	0.5967	0.0986	0.4041	0.6027	0.7983	0.6432
	γ	1.0	1.0639	0.0878	0.8412	1.0734	1.1998	1.0157
	μ	3.0	3.1011	0.1428	2.8433	3.1214	3.3861	2.9560
*n* = 50	τ	0.6	0.6389	0.1510	0.3696	0.6456	0.8706	0.5258
	γ	1.0	1.1032	0.1707	0.8495	1.0739	1.4655	0.9629
	μ	3.0	2.9777	0.2014	2.6674	2.9620	3.2630	2.9674
*n* = 20	τ	0.6	0.6571	0.2084	0.3139	0.6539	0.9103	0.5216
	γ	1.0	1.0203	0.1147	0.8313	1.0037	1.2143	1.1558
Scenario 3 (*p* = 2)								
	μ	2.0	2.0865	0.1512	1.8514	2.0701	2.4726	2.0578
*n* = 500	τ	1.0	1.0185	0.3038	0.3688	0.9740	1.7048	0.8468
	γ	−1.0	−0.9515	0.3780	−1.5384	−1.0019	−0.0617	−1.1953
	μ	2.0	2.0976	0.1591	1.8746	2.0766	2.4898	1.9338
*n* = 100	τ	1.0	1.0518	0.1968	0.7561	1.0252	1.4816	0.9890
	γ	−1.0	−0.9717	0.3878	−1.5099	−1.0616	0.0234	−0.9795
	μ	2.0	2.0671	0.1407	1.8745	2.0492	2.3671	1.9274
*n* = 50	τ	1.0	1.0503	0.1853	0.7767	1.0282	1.4259	0.9899
	γ	−1.0	−1.0243	0.3483	−1.5132	−1.0963	−0.3674	−0.9612
	μ	2.0	2.0406	0.1480	1.8427	2.0338	2.3473	1.9457
*n* = 20	τ	1.0	1.0650	0.1608	0.8113	1.0457	1.3459	0.9899
	γ	−1.0	−1.0472	0.4333	−1.5279	−1.1187	−0.0711	−0.9801
Scenario 4 (*p* = 2)								
	μ	3.0	3.1034	0.1231	2.7620	3.1036	3.3263	3.1364
*n* = 500	τ	0.6	0.5714	0.1592	0.2248	0.5508	0.9029	0.5621
	γ	1.0	0.9923	0.0837	0.8458	0.9904	1.1678	1.0903
	μ	3.0	3.2601	0.0829	3.1212	3.2432	3.4170	3.2175
*n* = 100	τ	0.6	0.5967	0.1442	0.4130	0.5747	0.8620	0.5985
	γ	1.0	0.9095	0.0592	0.8041	0.9173	0.9903	0.9261
	μ	3.0	3.1193	0.0888	2.9994	3.1142	3.2818	3.0141
*n* = 50	τ	0.6	0.6047	0.1597	0.4307	0.5747	0.8464	0.4968
	γ	1.0	1.0161	0.0861	0.9059	0.9945	1.1983	0.9820
	μ	3.0	2.9734	0.1287	2.8209	2.9589	3.2492	2.8303
*n* = 20	τ	0.6	0.6150	0.2097	0.4322	0.5775	1.1058	0.4968
	γ	1.0	1.0066	0.0779	0.8978	0.9888	1.1682	0.9820
Scenario 3 (*p* = 3)								
	μ_1_	1.0	0.9512	0.0547	0.8766	0.9495	1.0569	0.9386
	μ_2_	2.0	2.0385	0.0635	1.9296	2.0296	2.1261	1.9755
*n* = 500	τ	2.0	1.9858	0.9318	0.5731	1.8519	4.3790	1.8534
	γ_1_	1.0	0.9081	0.0112	0.8836	0.9097	0.9257	0.9123
	γ_2_	−1.0	−0.9724	0.5095	−1.0492	−0.9856	−0.8903	−1.0477
	μ_1_	1.0	0.9996	0.0347	0.9473	0.9970	1.0729	0.9877
	μ_2_	2.0	2.0020	0.0329	1.9378	2.0016	2.0690	2.0731
*n* = 100	τ	2.0	2.1327	0.6247	1.5256	2.0795	2.6173	1.8732
	γ_1_	1.0	0.9092	0.0119	0.8845	0.9116	0.9284	0.9130
	γ_2_	−1.0	−0.9082	0.0113	−0.9257	−0.9091	−0.8835	−0.9173
	μ_1_	1.0	1.1198	0.0171	1.0921	1.1166	1.1533	1.1262
	μ_2_	2.0	2.1168	0.0157	2.0908	2.1146	2.1449	2.1096
*n* = 50	τ	2.0	2.0631	0.3130	1.5308	2.0340	2.6085	2.2340
	γ_1_	1.0	0.9089	0.0122	0.8809	0.9095	0.9265	0.9126
	γ_2_	−1.0	−0.9073	0.0106	−0.9242	−0.9095	−0.8842	−0.9173
	μ_1_	1.0	0.8940	0.0244	0.8390	0.9081	0.9248	0.8927
	μ_2_	2.0	1.9098	0.0760	1.8415	1.8957	2.1224	1.8639
*n* = 20	τ	2.0	1.9192	0.3611	1.3785	1.8825	2.5855	1.8419
	γ_1_	1.0	0.9275	0.0109	0.9068	0.9259	0.9482	0.9248
	γ_2_	−1.0	−0.9282	0.0086	−0.9426	−0.9282	−0.9124	−0.9360

**Figure 2:**
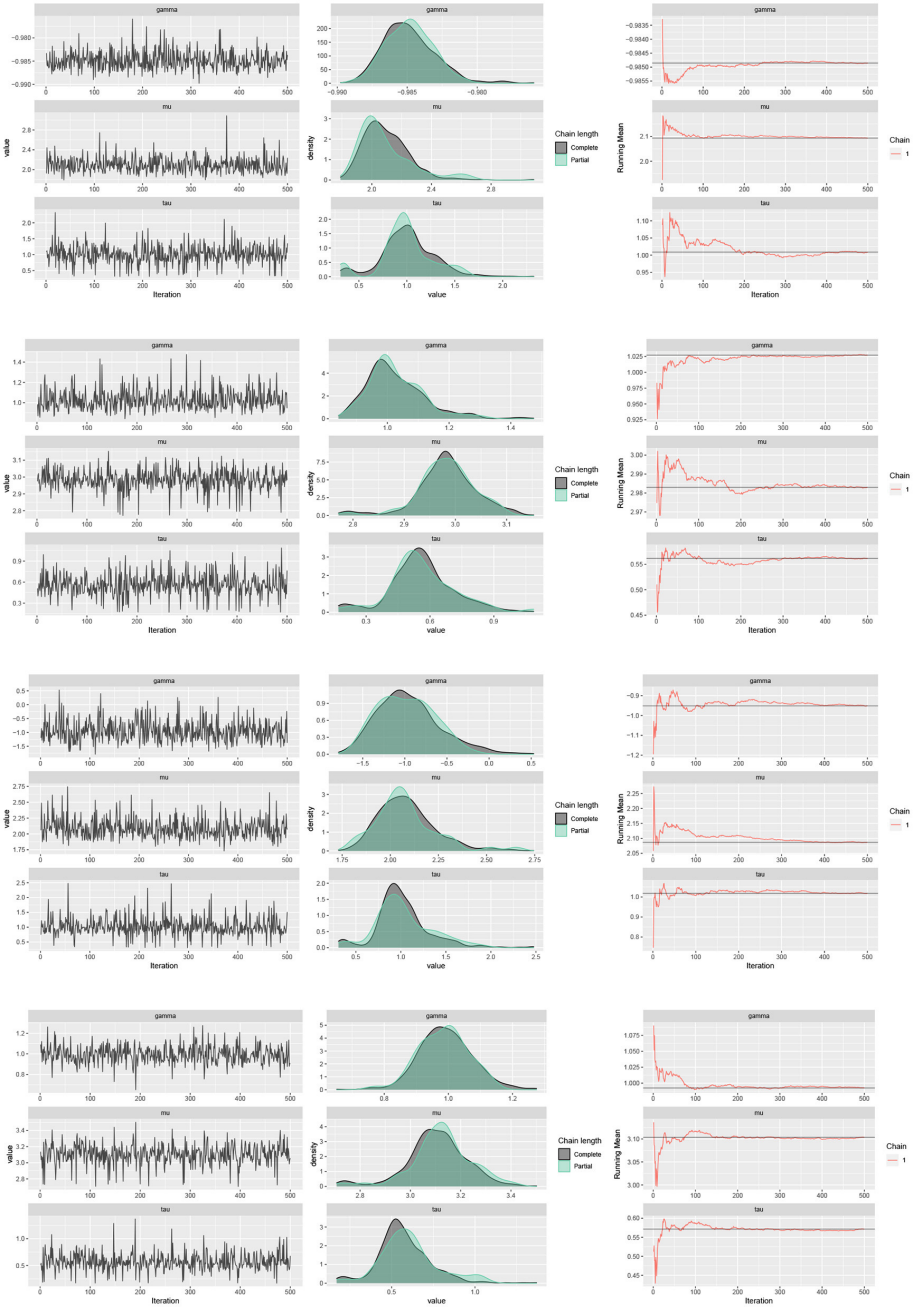
Traceplots, mean running and estimated posterior pdf plots of generated samples for (μ, τ, γ) in Table 1 for *p* = 2, *n* = 500 and scenario 1 (first row), scenario 2 (second row), scenario 3 (third row), and scenario 4 (fourth row).

**Figure 3:**
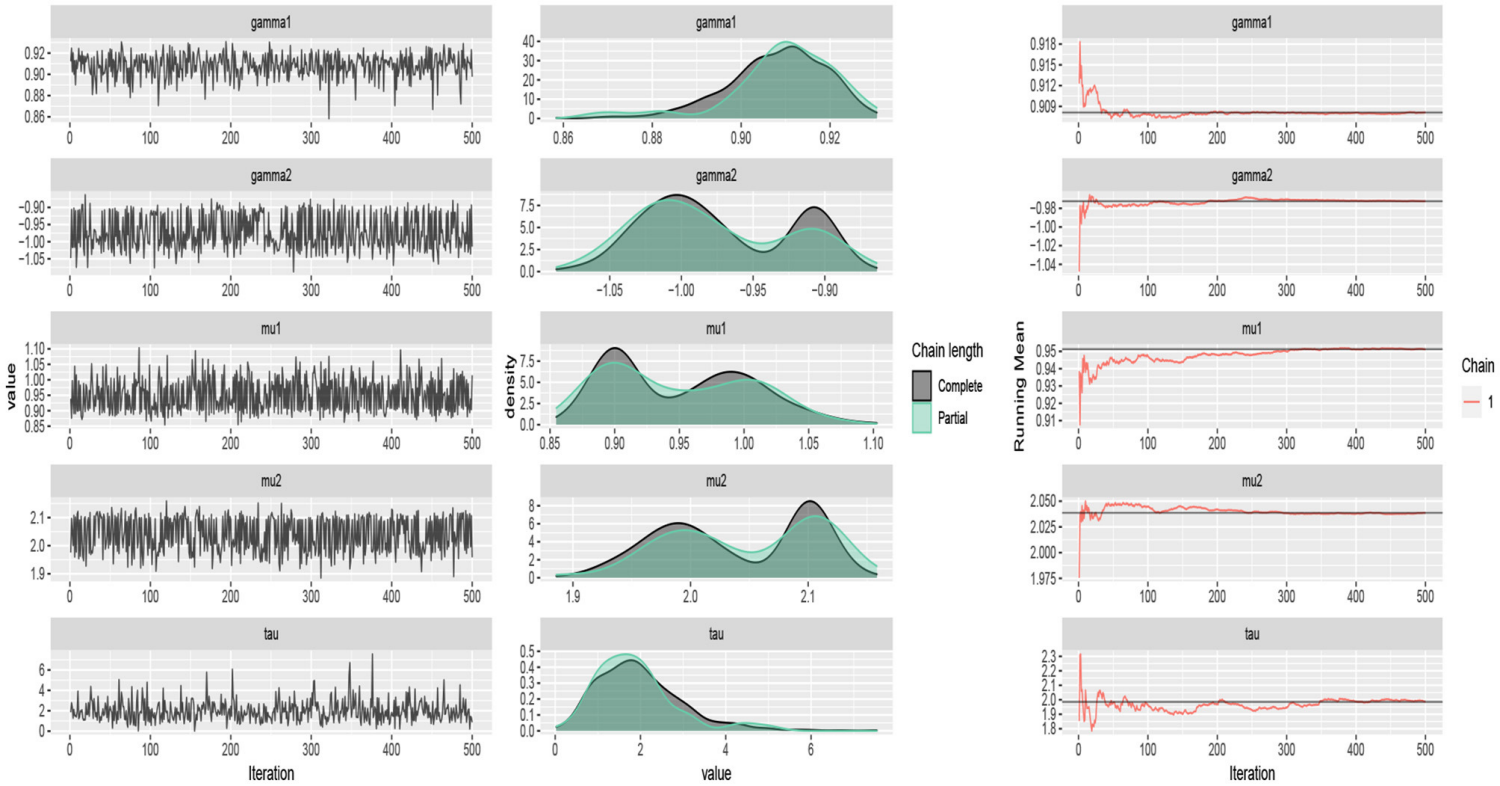
Traceplots, mean running and estimated posterior pdf plots of generated samples for (μ_1_, μ_2_, τ, γ_1_, γ_2_) in Table 1 for *p* = 3 and *n* = 500.

The traceplots of the generated samples from the posteriors, the compare-partial plots and the running mean plots are shown in Figure 2 (*p* = 2) and Figure 3 (*p* = 3) for each of the scenarios and *p* = 2 and 3 using the ggmcmc package in *R* (Fernández-i-Marın, 2016). A traceplot is an essential plot for evaluating convergence and diagnosing chain problems. It shows the time series of the sampling process and the expected outcome is to get a traceplot that looks completely random. A compare-partial plot provides overlapped kernel density plots that compare the last part of the chain (the last 10% of the values, in green) with the whole chain (in black). Ideally, the initial and final parts of the chain have to be sampling in the same target distribution, so the overlapped densities should be similar. In addition to the traceplots, the running mean plot of the chains is very useful to find within-chain convergence issues. A time series of the running mean of the chain allows to check whether the chain is slowly or quickly approaching its target distribution. The expected output is a line that quickly approaches the overall mean. Figures 2, 3 confirm the convergence of the chains and show that the modified Gibbs sampler recovers the values that actually come from the target posterior distributions.

**Figure 4:**
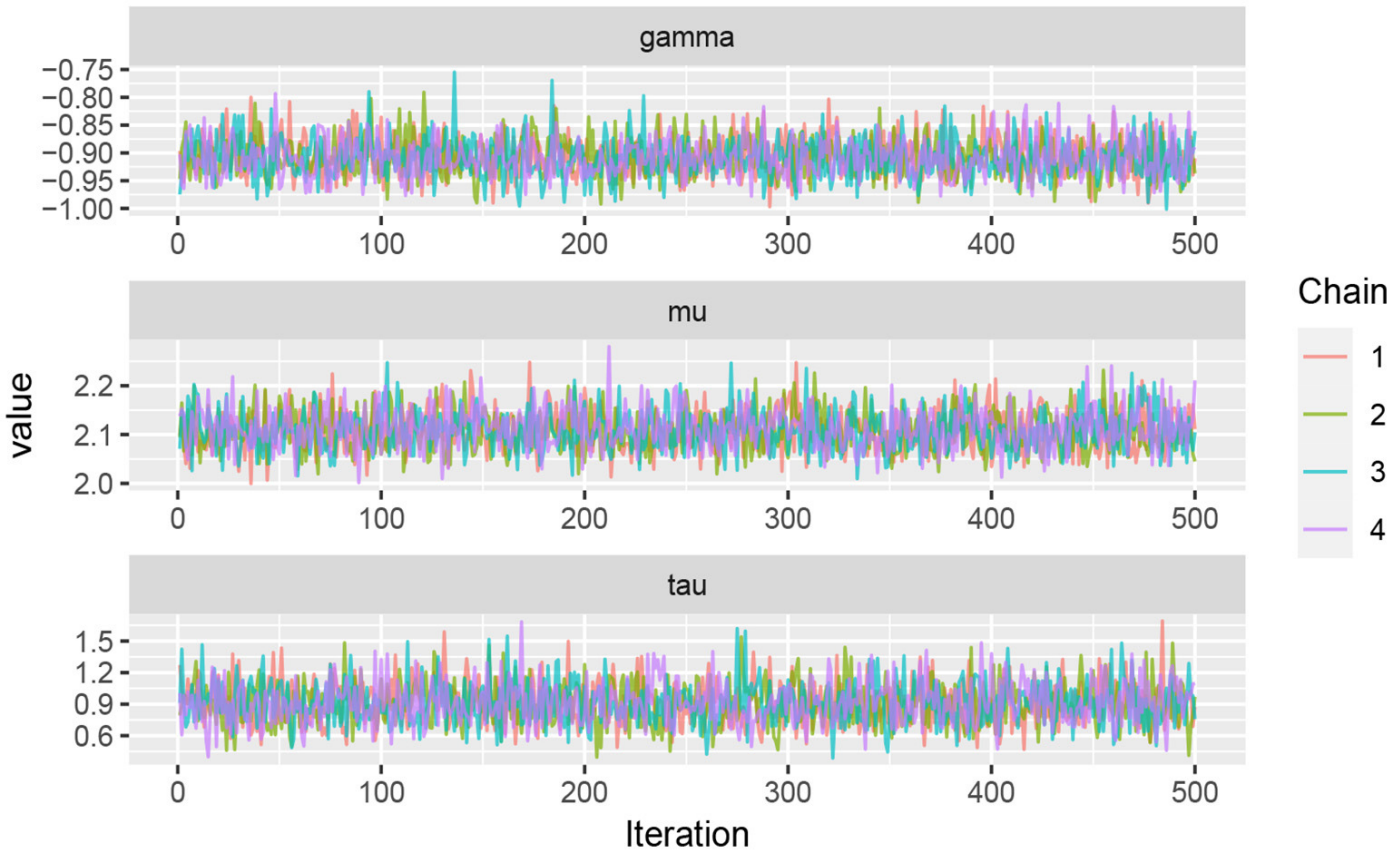
Traceplots of generated samples of size *n* = 500 from four parallel chains for (μ, τ, γ) based on scenario 3 (*p* = 2) in Table 1.

Running multiple independent chains in parallel is necessary to access the representativeness of the chains. If the multiple chains are not well mixed, the convergence of the chains is suspected (Kruschke, 2014; Vehtari et al., 2021). Therefore, four independent chains were run in parallel for scenario 3 (*p* = 2) in Table 1 to make the inference more robust and reliable. The results are shown in Figure 4 which confirm the convergency.

**Figure 5:**
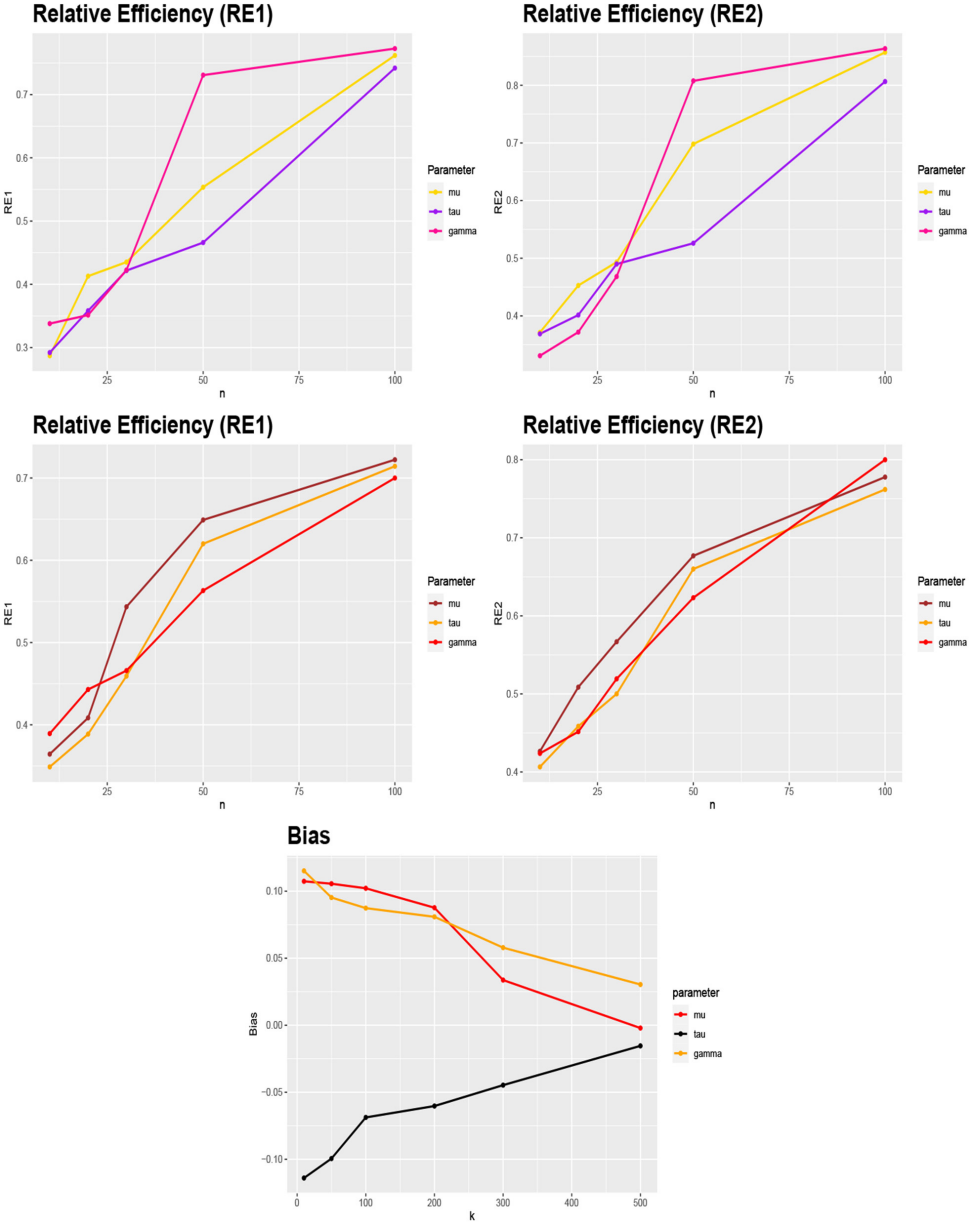
**(Top)** the RE of the Bayes estimates and MLEs of μ, τ, and γ vs. the sample size *n* for scenario 2. **(Middle)** the RE of the Bayes estimates and MLEs of μ, τ, and γ vs. the sample size *n* for scenario 3. **(Bottom)** the biases of the Bayes estimates of (μ, τ, γ) under the squared error loss function for scenario 3, *p* = 2, *n* = 100 and different values of *k* = 10, 50, 100, 200, 300, 500.

To compare the efficiency of Bayes estimates with respect to the maximum likelihood estimations (MLE), the mean squared errors (MSE) of MLEs and Bayes estimates of parameters under the squared error and absolute error loss functions were obtained for scenario 2 and 3 and *n* = 10, 20, 30, 50, 100 using a Monte Carlo simulation with 500 replications. Then, the relative efficiency (RE) was computed as


$$\begin{array}{l} RE_{1}=\frac{MSE\left(\hat{\Omega }\right)}{MSE\left(\tilde{\Omega }\right)},RE_{2}=\frac{MSE\left(\hat{\hat{\Omega }}\right)}{MSE\left(\tilde{\Omega }\right)}, \end{array}$$

where $\tilde{\Omega }$ is the MLE of **Ω** = (μ, τ, γ) and $\hat{\Omega }$ and $\hat{\hat{\Omega }}$ are the Bayes estimates of **Ω** under the squared error and absolute error loss functions, respectively. The results are shown in Figure 5 for scenario 2 (top) and scenario 3 (middle) and μ = 3, τ = 0.6, γ = 1.

From Figure 5 (top and middle) the following general conclusions can be observed:

- Our proposed Bayesian approach provides more accurate estimates for parameters in comparison with the maximum likelihood method for small values of *n*.
- The obtained Bayes estimates under the squared error loss function have less MSE than the estimates based on absolute error loss function.
- By increasing *n*, our proposed Bayesian approach has a similar performance as the maximum likelihood method.

Finally, to investigate the rule of *k* in Algorithm 1, the biases of the Bayes estimates of (μ, τ, γ) under the squared error loss function were obtained for scenario 3 (*p* = 2) in Table 1, *n* = 100 and different values of *k* = 10, 50, 100, 200, 300, 500 using a Monte Carlo simulation with 500 replications. The results are shown in Figure 5 (bottom) which demonstrate that, by increasing *k*, the bias tends to zero and thus, the accuracy of estimates increases.

## 6. Data application

**Figure 6:**
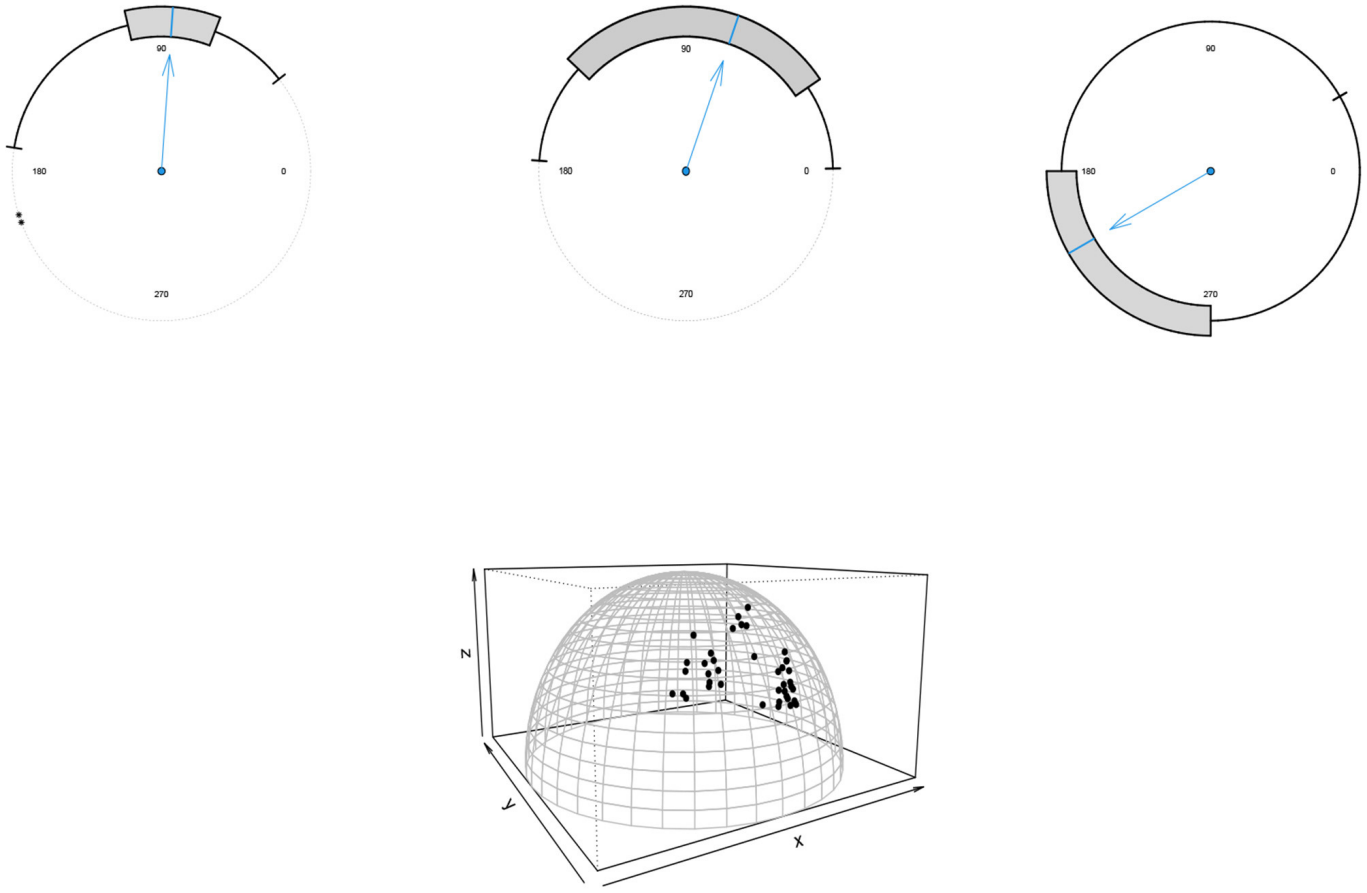
**(Top)** the boxplots of the movement of blue periwinkles (left), long-axis orientations of feldspar laths (middle) and thunder at Kew (right) datasets. **(Bottom)** the scatter plot of the household expenditures dataset.

In what follows, the proposed Bayesian approach's performance for *p* = 2 is demonstrated through three datasets with different sizes *n* = 31, 60, 725 with the skew-von Mises distribution in (6) as assumed model. The circular boxplots (Buttarazzi et al., 2018) of the datasets are shown in Figure 6 (top) and confirm the skew pattern of the datasets.

**Figure 7:**
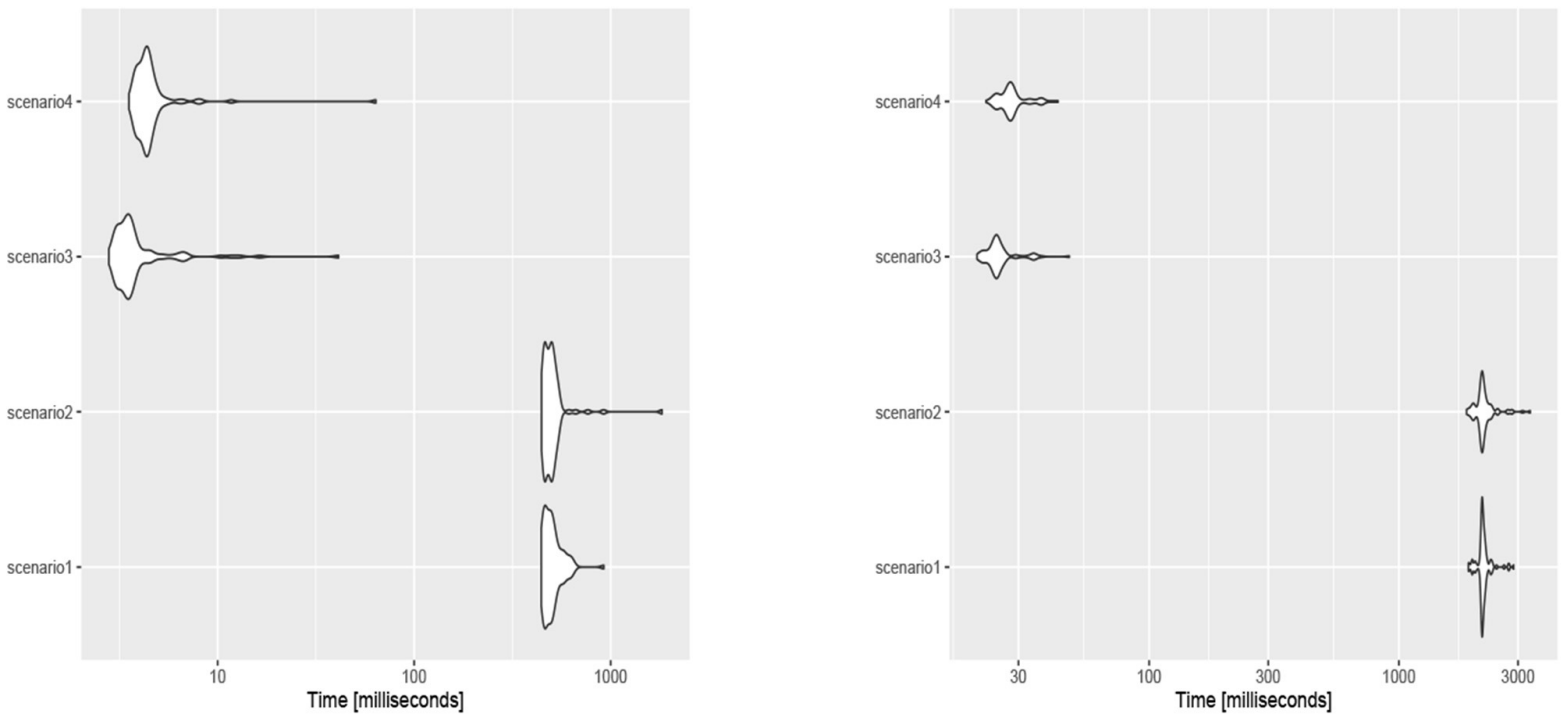
The execution time (in miliseconds) for generating samples of size *n* = 10 (left) and *n* = 50 (right) from the posterior density functions of each scenario.

The obtained results in section 5 show all the assumed scenarios provide accurate estimates for the parameters. However, based on the obtained results in section 5 with the WIM, we propose scenario 3 or 4 for obtaining the Bayes estimates to avoid time intensive computations when the sample size is not small (see Figure 7). The justification is that scenarios 1 and 2 need the slice sampler in Algorithm 1 to generate samples from the joint prior (10). Therefore, the Bayes estimates of the parameters μ, τ, and γ were obtained based on scenario 1 for the movement of blue periwinkles dataset (*n* = 31); scenarios 3 and 4 for the long-axis orientations of feldspar laths (*n* = 60) and the thunder at Kew (*n* = 725) datasets, respectively. See below for the description of said datasets.

For *p* = 3, a dataset of size *n* = 40 including the expenditures of households is considered. Figure 6 (bottom) shows the scatter plot of the data. For all the datasets to obtain the Bayes estimates we generated samples of size *N* = 1, 000 from the posterior distributions using Algorithm 1 with a burn-in period of 10,000 and *k* = 500.

In what follows, we shall describe the individual datasets in detail. Since the conclusion is more or less the same for all *p* = 2 settings, we already write it down here. It is observed that the proposed Bayesian approach with the skew von Mises distribution as the underlying model provides a good fit to the datasets. Generally, the obtained estimates based on the squared error and absolute error loss functions are more accurate.

### 6.1. Movement of Blue Periwinkles

A real dataset including the movement directions of 31 blue periwinkles, *Nodilittorina unifasciata*, in degrees is considered (Fisher, 1995). The data was collected from a series of experiments which were done on the distances and directions that small blue periwinkles moved after the transplantation to downshore at a specific height where they live normally. The test of Pewsey (2002) confirms that the underlying distribution for this dataset is asymmetric (p-value = 0.0000). This test is defined based on the sample second sine moment ${\overset{-}{b}}_{2}=\frac{1}{n}\overset{n}{\underset{i=1}{\sum }}\sin2\left(\theta_{i}-\overset{-}{\theta }\right)$ where $\overset{-}{\theta }$ is the sample mean direction. The large values of $|\frac{{\overset{-}{b}}_{2}}{\sqrt{\hat{var}\left({\overset{-}{b}}_{2}\right)}}|$ compared with the quantiles of the standard normal distribution lead to the rejection of symmetry. For more details see Pewsey (2002).

**Figure 8:**
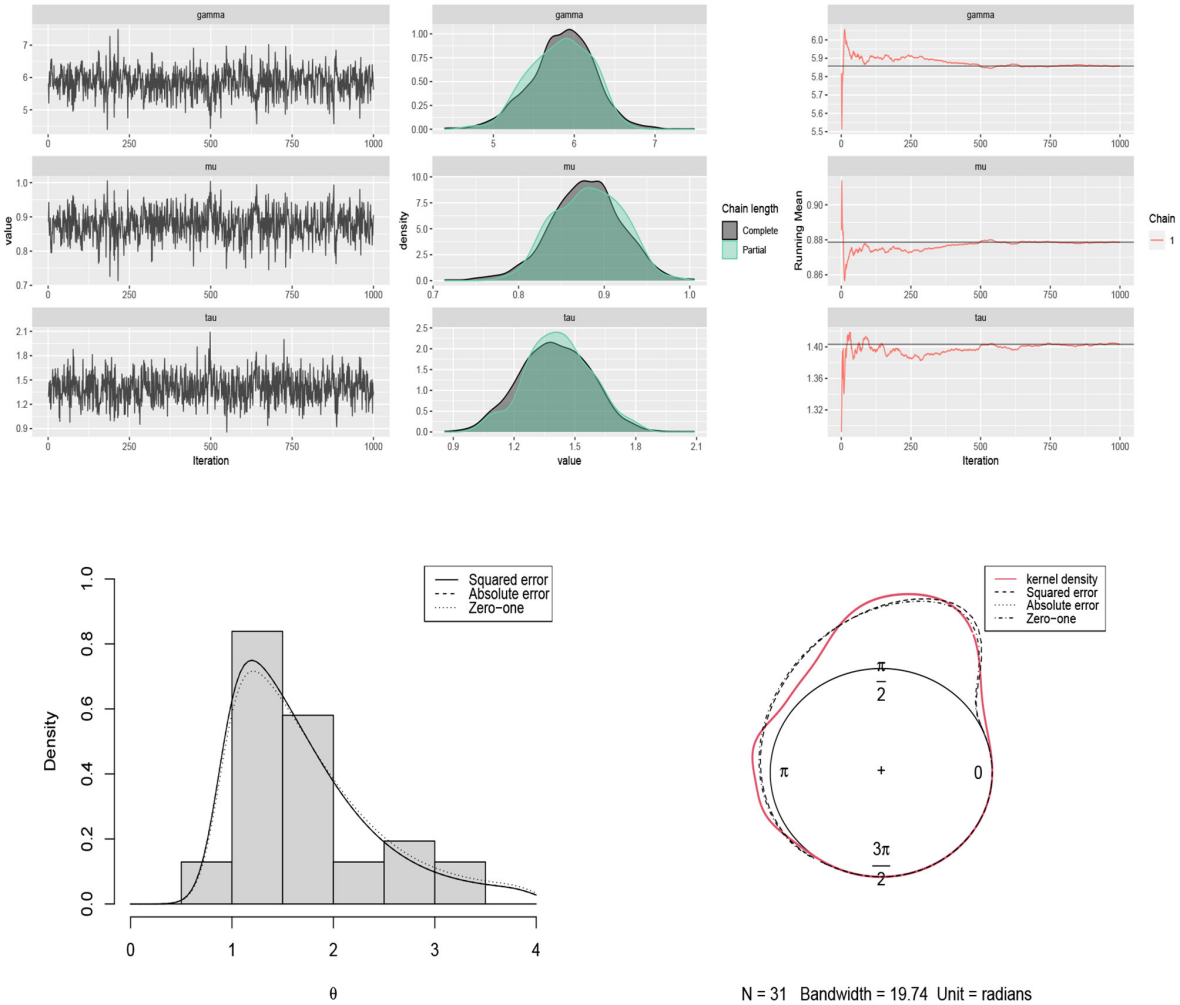
**(Top)** traceplots, mean running and estimated posterior pdf plots of generated samples for (μ, τ, γ) in Table 2 for the movement of blue periwinkles dataset. **(Bottom)** the histogram and kernel density plot of the data related to the movement of blue periwinkles and the fitted curves under different loss functions.

The Bayes estimates of parameters are obtained by using scenario 1 based on squared error, absolute error and zero-one loss functions. The results are summarized in Table 2. The traceplots of generated samples from the posterior, the compare-partial and mean running plots are shown in Figure 8 (top). The kernel density plot and histogram of the data along with the fitted curves under different loss functions are shown in Figure 8 (bottom).

**Table 2:** Bayes estimates of parameters based on scenario 1 with prior parameters μ_0_ = 2, ζ = 2, η = 1, ξ = 5, and σ = 2 for the movement of blue periwinkles dataset.

**Parameter**	**Mean**	**Mode**	**sd**	***Q***_**1**_	**Median**	***Q***_**3**_
μ	0.8786	0.8854	0.04155	0.7952	0.8785	0.9574
τ	1.4033	1.2918	0.1729	1.0746	1.4021	1.7409
γ	5.8574	5.8183	0.3974	5.0180	5.8851	6.5687

### 6.2. Long-Axis Orientations of Feldspar Laths

**Figure 9:**
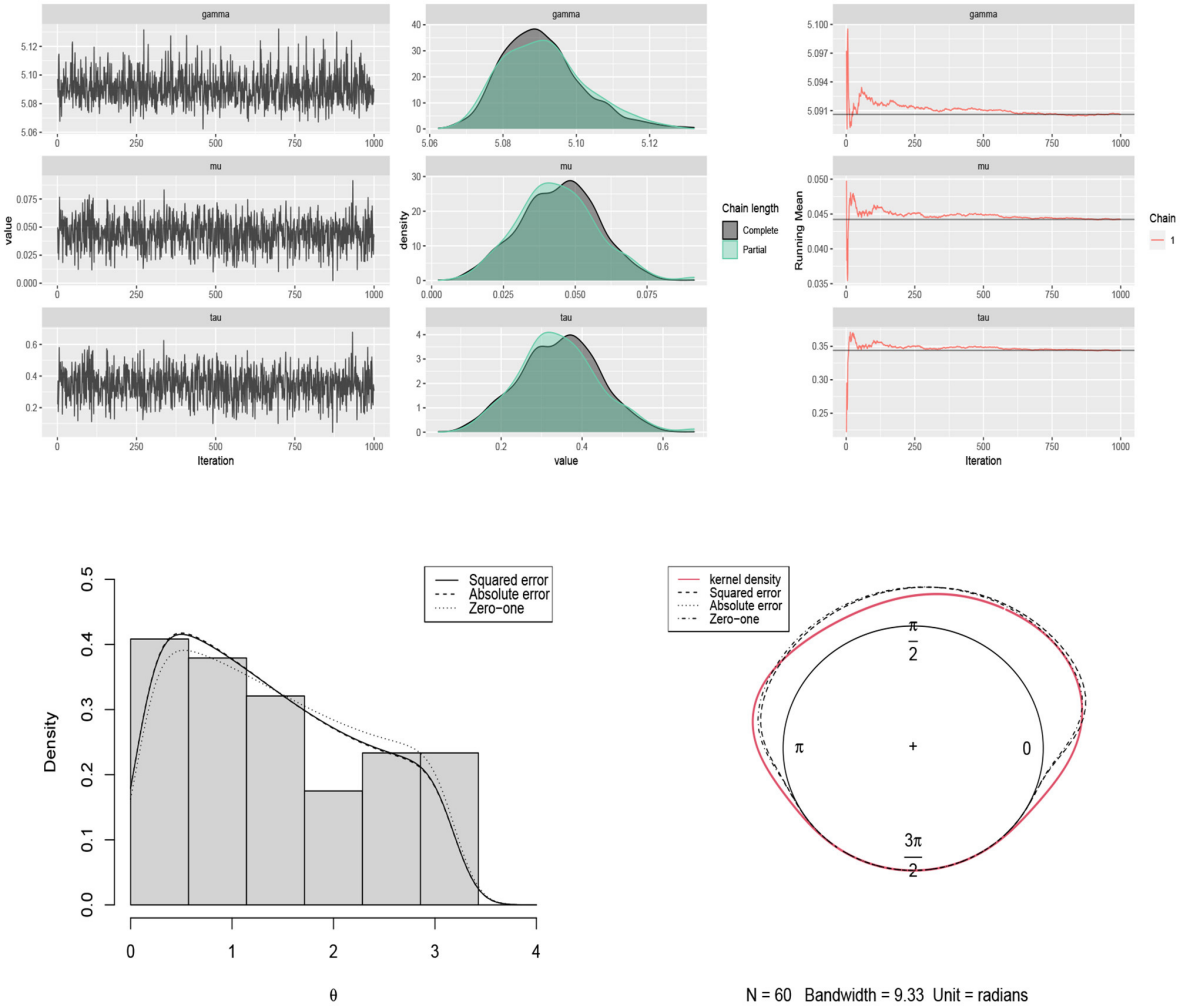
**(Top)** traceplots, mean running and estimated posterior pdf plots of generated samples for (μ, τ, γ) in Table 3 for the long-axis orientations of feldspar laths dataset. **(Bottom)** the histogram and kernel density plot of the data related to the long-axis orientations of feldspar laths and the fitted curves under different loss functions.

Another dataset including the measurements of long-axis orientation of 60 feldspar laths in basalt (Fisher, 1995) is considered. The symmetry test of Pewsey (2002) confirms the skew pattern of the data in Figure 6 (p-value = 0.0000). The Bayes estimates of parameters are obtained by using scenario 3 based on squared error, absolute error and zero-one loss functions. The results are summarized in Table 3. The traceplots of generated samples from the posterior, the compare-partial, and the mean running plots are shown in Figure 9 (top). The histogram and kernel density plot of the data and the fitted curves under different loss functions are shown in Figure 9 (bottom).

**Table 3:** Bayes estimates of parameters based on scenario 3 with prior parameters μ_0_ = 0, τ_0_ = 8, α = 5, β = 2, ξ = 3, σ = 1 for the long-axis orientations of feldspar laths dataset.

**Parameter**	**Mean**	**Mode**	**sd**	***Q***_1_	**Median**	***Q***_3_
μ	0.0442	0.0497	0.0134	0.0180	0.0451	0.0697
τ	0.3438	0.2616	0.0970	0.1571	0.3504	0.5301
γ	5.0906	5.0972	0.0109	5.0729	5.0894	5.1158

### 6.3. Thunder at Kew

**Figure 10:**
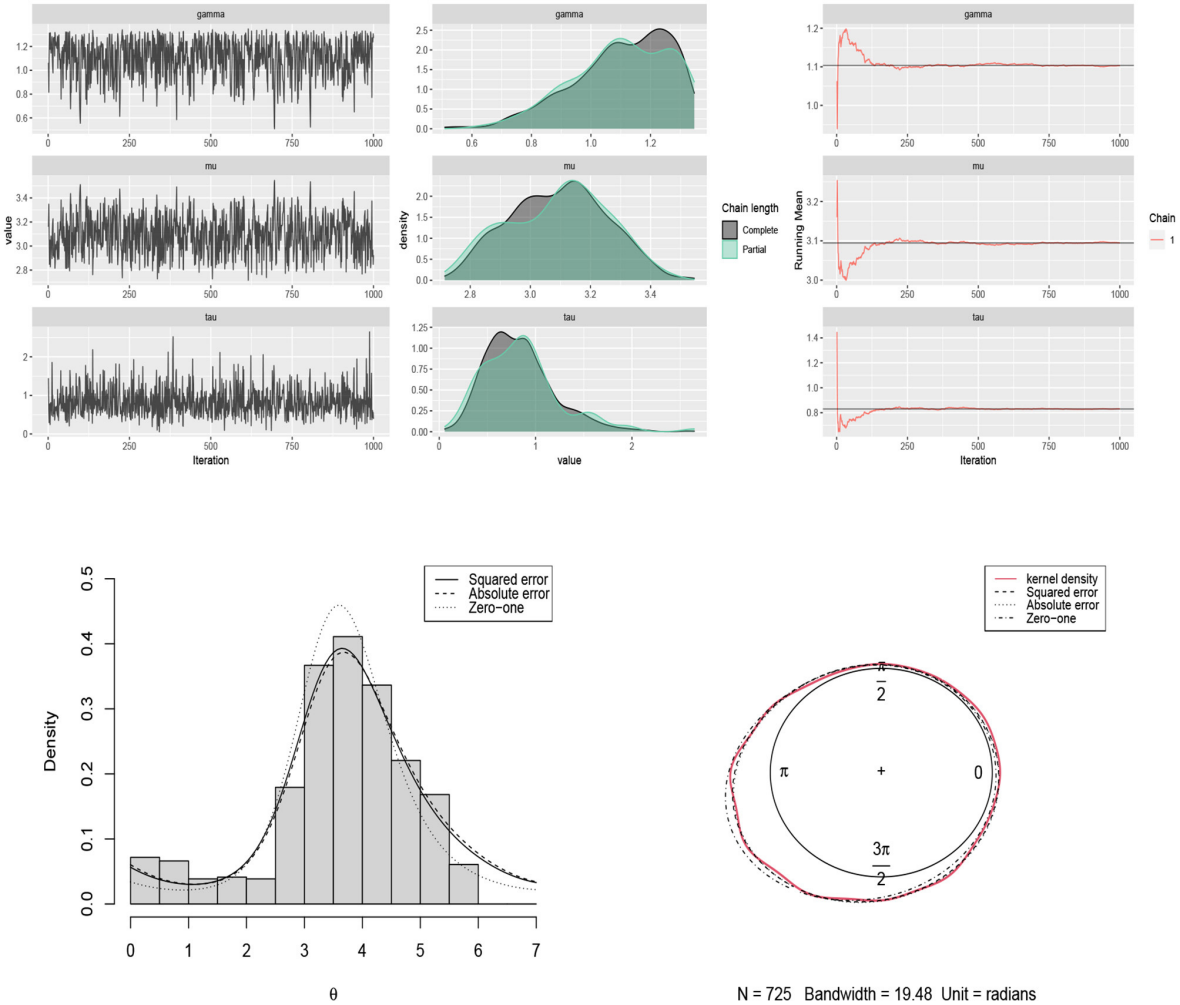
**(Top)** traceplots, mean running and estimated posterior pdf plots of generated samples for (μ, τ, γ) in Table 4 for the thunder at Kew dataset. **(Bottom)** the histogram and kernel density plot of the data related to the thunder at Kew and the fitted curves under different loss functions.

A grouped frequency data set consisting of 725 observations about the number of times that thunder was heard at Kew (England) during each two hourly interval of the day in the summers of 1910–1935 is considered (Mardia, 1975). In this case, each 15° on the circle represents 1 h. According to the test of Pewsey (2002), the underlying distribution for this data set is not symmetric (p-value = 0.0000). The Bayes estimates of parameters are obtained by using scenario 4 based on squared error, absolute error and zero-one loss functions. The results are summarized in Table 4. The traceplots of generated samples from the posterior, the compare-partial and mean running plots are shown in Figure 10 (top). The histogram and kernel density plot of the data and the fitted curves under different loss functions are shown in Figure 10 (bottom).

**Table 4:** Bayes estimates of parameters based on scenario 4 with prior parameters μ_0_ = 3, τ_0_ = 2, α = 5, β = 6, ξ = 0.5, σ = 0.5, and λ = −2 for the thunder at Kew dataset.

**Parameter**	**Mean**	**Mode**	**sd**	***Q***_1_	**Median**	***Q***_3_
μ	3.0943	3.1601	0.1598	2.8035	3.1032	3.3948
τ	0.9675	1.2178	0.3597	0.2863	0.7868	1.6630
γ	0.9179	1.0629	0.1610	0.7402	1.1234	1.3237

### 6.4. Household Expenditures

**Figure 11:**
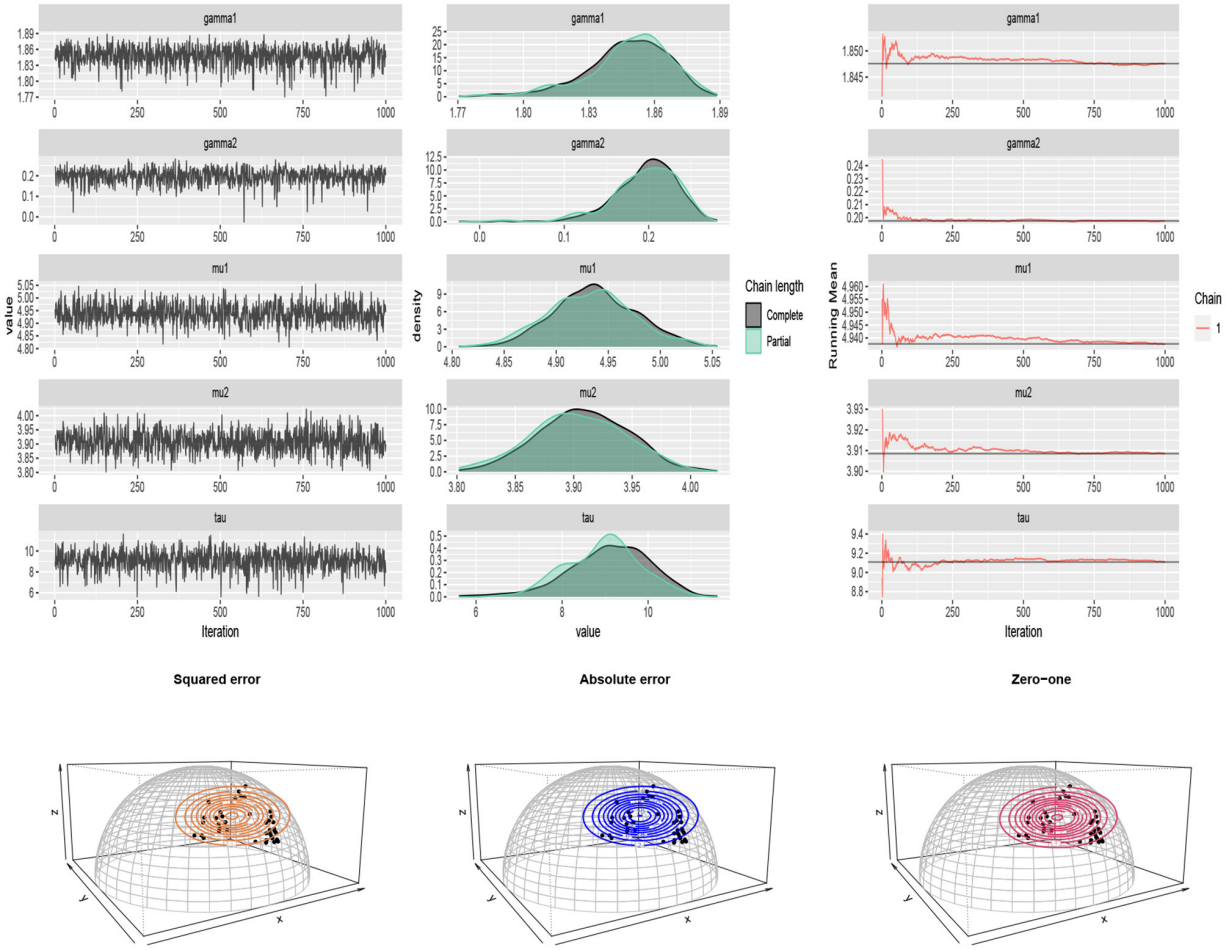
**(Top)** traceplots, mean running and estimated posterior pdf plots of generated samples for (μ_1_, μ_2_, τ, γ_1_, γ_2_) in Table 5 for the household expenditures dataset. **(Bottom)** the scatter plot of the household expenditures dataset and the contour plot of the fitted distribution under different loss functions.

For *p* = 3, a sub data from the dataset available in the HSAUR2 package (Everitt and Hothorn, 2017) in *R* is considered. The entire data was collected from a survey on household expenditures in four commodity groups of housing, food, goods, and service. It includes the expenses of 20 single men and 20 single women. We considered variables housing, food, and service and normalized. After applying cosine transformation (5), the *SFvML* was fitted on the data and the Bayes estimates of the parameters were obtained. The results are summarized in Table 5. The traceplots of generated samples from the posterior and the compare-partial and mean running plots are shown in Figure 11 (top). The scatter plot of the data and the contour plot of the fitted distribution under different loss functions are shown in Figure 11 (bottom).

**Table 5:** Bayes estimates of parameters based on scenario 3 with prior parameters μ_0_1__ = 3, τ_0_1__ = 2, μ_0_2__ = 3, τ_0_2__ = 4, α = 20, β = 1, ξ_1_ = 0, σ_1_ = 3, ξ_1_ = 0, and σ_1_ = 2 for the household expenditures dataset.

**Parameter**	**Mean**	**Mode**	**sd**	***Q***_1_	**Median**	***Q***_3_
μ_1_	4.9376	4.9550	0.0392	4.8634	4.9362	5.0156
μ_2_	3.9085	3.9300	0.0384	3.8327	3.9080	3.9828
τ	9.1075	8.9427	0.9499	6.9295	9.1673	10.7127
γ_1_	1.8475	1.8412	0.0189	1.8019	1.8490	1.8773
γ_2_	0.1974	0.2449	0.0365	0.1184	0.2008	0.2550

## 7. Conclusion

Since the assumption that data is rotationally-symmetric is often rejected (Pewsey, 2002; Ley and Verdebout, 2014; Ameijeiras-Alonso and Ley, 2020; Ameijeiras-Alonso et al., 2021), in this paper, we have presented a Bayesian analysis for the skew-rotationally-symmetric FvML distribution. For the first time in Bayesian analysis of directional data the impact of the proposed priors in the set up has been compared using the Wasserstein Impact Measure. Using this measure can give guidance to the practitioner to avoid computationally intensive priors if a simpler prior has similar impact. An algorithm has been used based on modified Gibbs sampling and weighted bootstrap resampling for generating samples from posterior distributions. This coming together of Bayesian methods and skew distributions in the directional domain promises new research interest.

## Data Availability Statement

All relevant references for data are contained within the article.

## Author Contributions

All authors listed have made a substantial, direct, and intellectual contribution to the work and approved it for publication.

## Funding

This work was based upon research supported in part by the Visiting Professor programme, University of Pretoria and the National Research Foundation (NRF) of South Africa, SARChI Research Chair UID: 71199; Ref.: IFR170227223754 grant No. 109214; Ref.: SRUG190308422768 grant No. 120839, and DSI-NRF Centre of Excellence in Mathematical and Statistical Sciences (CoE-MaSS), South Africa. The opinions expressed and conclusions arrived at are those of the authors and are not necessarily to be attributed to the CoE-MaSS or the NRF. Christophe Ley's research is supported by the FWO Krediet aan Navorsers grant with reference number 1510391N.

## Conflict of Interest

The authors declare that the research was conducted in the absence of any commercial or financial relationships that could be construed as a potential conflict of interest.

## Publisher's Note

All claims expressed in this article are solely those of the authors and do not necessarily represent those of their affiliated organizations, or those of the publisher, the editors and the reviewers. Any product that may be evaluated in this article, or claim that may be made by its manufacturer, is not guaranteed or endorsed by the publisher.
